# Supplementary material for: Copy-number variants and polygenic risk for intelligence confer risk for autism spectrum disorder irrespective of their effects on cognitive ability
Source: Front Psychiatry. 2024 May 1;15:1369767. doi: 10.3389/fpsyt.2024.1369767 (PMC11094536; doi:10.3389/fpsyt.2024.1369767)
Supplement: Supplementary file 1 [file DataSheet_1.pdf]

Copy-number variants and polygenic risk for intelligence confer risk for autism spectrum disorder irrespective of their effects on cognitive ability

Supplementary Notes

Role of deletions and duplications encompassing highly intolerant genes in modulating the impact of PRS-IQ on ASD risk and cognitive ability

To further investigate how CNVs in highly intolerant genes may modulate the impact of PRS-IQ on ASD risk and cognitive ability, we stratified all subjects with available cognitive data into three groups: 1–subjects carrying CNVs in two or more highly constrained genes; 2–subjects carrying a CNV in one highly constrained gene, and; 3–subjects that carried no CNVs in highly constrained genes. A highly constrained gene was defined as having a LOEUF score ≥0.35[29]. This stratification according to CNV carrier status was performed for deletions and duplications separately. The number of cases with ASD and extrafamilial controls in each CNV carrier category is detailed in Table ST4.

We then estimated the impact of PRS-IQ on ASD risk and cognitive ability using the CNV carrier status of the individuals (i.e.: carrying none, one, or two or more deletions or duplications in haploinsufficient genes) as a moderator variable as follows:

$$logit(ASD) \sim (DEL_{CNV\ carrier\ status} \times PRS_{IQ}) + (DUP_{CNV\ carrier\ status} \times PRS_{IQ}) + cognitive\ ability$$

$$lm(cognitive\ ability) \sim (DEL_{CNV\ carrier\ status} \times PRS_{IQ}) + (DUP_{CNV\ carrier\ status} \times PRS_{IQ}) + ASD$$

Cognitive ability and ASD case status were used as covariates in the logistic and linear regression models, respectively. Both models included sex as a covariate.

The *sim_slopes* function from the “interactions” R package was used to extract the effect of PRS-IQ on ASD risk and cognitive ability for the three CNV carrier status outcomes. The results were visualized using the *interaction_plot* function from the “interactions” R package for the two ASD risk and cognitive ability models. PRS-IQ and CNV carrier status were specified as the predictor and moderator variables, respectively. The impact of PRS-IQ on ASD risk and cognitive ability, as moderated by CNV carrier status, was performed for deletions and duplications separately.

To further understand the relationship between CNV carrier status, PRS-IQ, and cognitive ability, we ran the following regressions that explored the three-way interaction between the variables:

$$logit(ASD) \sim (DEL_{CNV\ carrier\ status} \times PRS_{IQ} \times cognitive\ ability) + (DUP_{CNV\ carrier\ status} \times PRS_{IQ} \times cognitive\ ability)$$

$$lm(cognitive\ ability) \sim (DEL_{CNV\ carrier\ status} \times PRS_{IQ} \times ASD) + (DUP_{CNV\ carrier\ status} \times PRS_{IQ} \times ASD)$$

Here, the logistic and linear regression models used cognitive ability and ASD diagnostic status as secondary moderator variables, respectively. Both models included sex as a covariate.

The logistic regression model assessed the interaction between PRS-IQ and CNV carrier status on ASD risk depending on the level of cognitive ability of the subject. Using PRS-IQ as the predictor and CNV carrier status as the moderator, the *sim_slopes* function used cognitive ability as its second moderator. As cognitive ability is a continuous variable, the estimate of PRS-IQ (slope of the focal predictor) on ASD risk for each CNV carrier status was determined according to the mean and ±1 standard deviation values of this moderator. For the linear regression, the estimate of PRS-IQ on cognitive ability for each CNV carrier status was evaluated across both levels of the ASD status moderator (i.e.: case or extrafamilial control) separately. These interactions were visualized using the *interact_plot* function from the "interactions" R package.

While we lack the statistical power to validate these interactions, we present an approach to assess the relationship between PRS-IQ and deletions and duplications in highly intolerant genes in modulating ASD risk and cognitive ability. Our preliminary results suggest that in the absence of deleted or duplicated highly intolerant genes, PRS-IQ has a positive linear impact on ASD risk and cognitive ability. Carrying one deletion in a highly intolerant gene attenuates the impact of PRS-IQ on ASD risk and cognitive ability. In the context of deletions and duplications of two or more intolerant genes, we no longer observed the effect of PRS-IQ on ASD risk and cognitive ability. The impact of PRS-IQ on ASD risk and cognitive ability was similarly attenuated among carriers of duplications in highly intolerant genes. The findings suggest that the impact of PRS-IQ on ASD risk and cognitive ability is attenuated in carriers of CNVs in highly intolerant genes, regardless of their cognitive ability (**Figures S9c, d**) and ASD case status (**Figure S9g, h**), respectively.

We posit that CNVs in highly intolerant genes and PRS-IQ may modulate these outcomes through a hypothesized framework detailed in **Figure S10**. Future studies with larger samples are warranted to validate this relationship.

Supplementary Figures

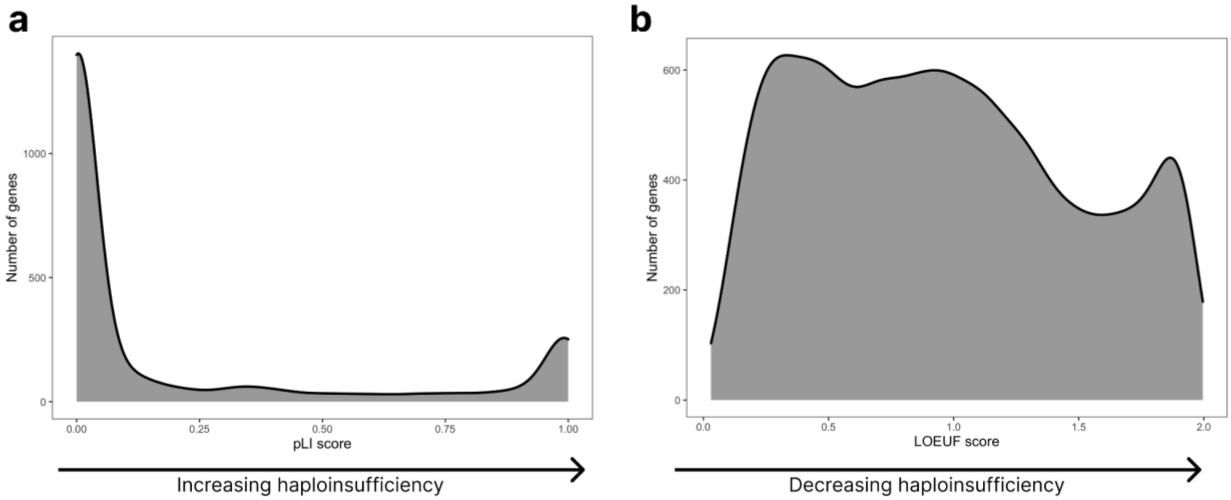

Figure S1. Distribution of haploinsufficiency scores across 19,197 genes.
a) The distribution of the probability of being loss-of-function intolerant (pLI) annotation. The bimodal distribution better captures the genome-wide burden of CNV risk across each individual. b) The distribution of the loss-of-function observed/expected upper bound fraction (LOEUF) annotation. The continuous metric facilitates the stratification of individuals according to their carrier status of CNVs encompassing haploinsufficient genes,

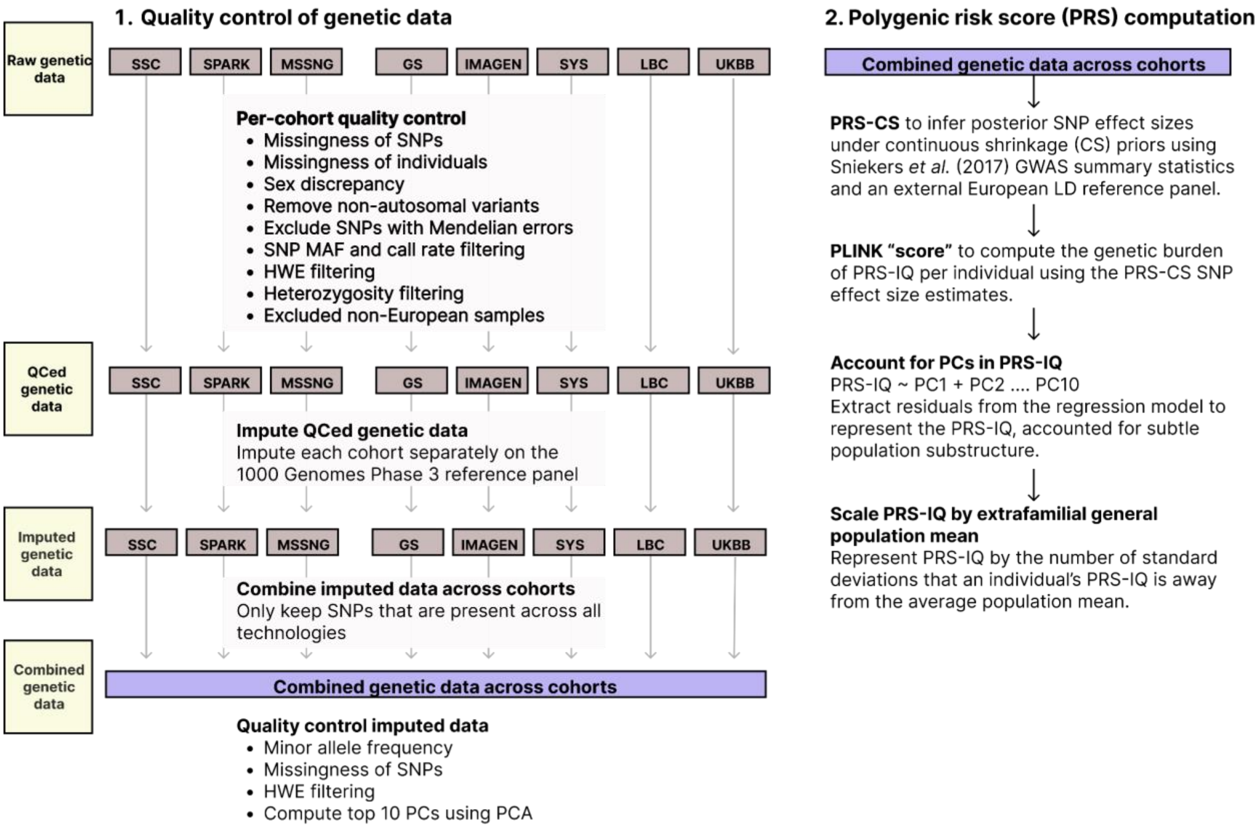

Figure S2. Methodological pipeline to compute and combine PRS-IQ data across eight cohorts. Description of each analysis step used to compute PRS-IQ across eight cohorts. To ensure that the PRS-IQ data across cohorts were comparable within each analysis group (cases with ASD, intrafamilial controls, and extrafamilial controls), we performed a one-way ANOVA and pairwise t.test (see: Tables ST2 and ST3). There was no significant difference in the mean PRS-IQ across cohorts following the final analysis step.

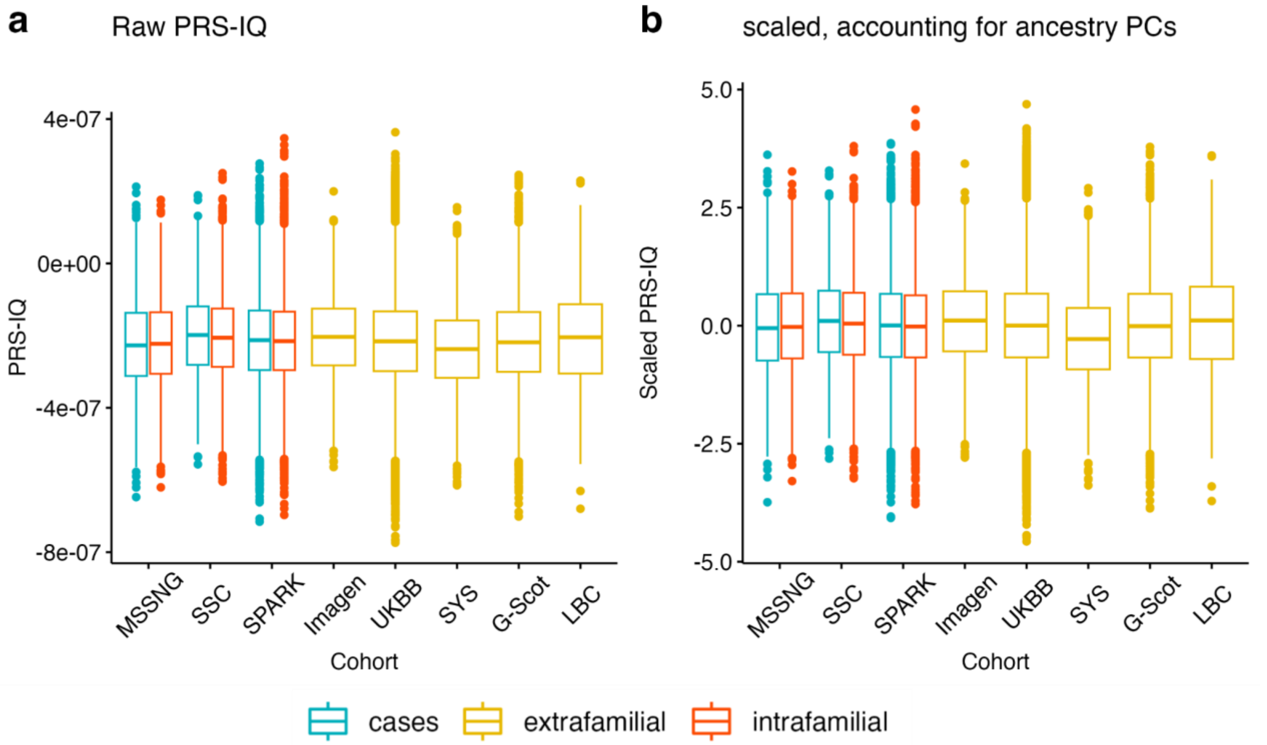

Figure S3. Distribution of PRS-IQ across cohorts in each analysis group.
The distribution of PRS-IQ across the different PRS computation steps: the "raw" PRS-IQ across all European samples (**a**), and; the scaled residuals after modelling PRS-IQ as a function of the top 20 ancestry PCs (**b**). We combined the imputed data such that only the variants that were present across all genotyping and sequencing technologies were retained. This step was important to reduce PRS-IQ biases due to batch effects. There was no difference in PRS-IQ between intrafamilial and extrafamilial controls (Figure 2c), which were derived from distinct cohorts. This validated our approach and findings that compared PRS-IQ between cases with ASD and extrafamilial controls. We used the Welch's ANOVA and Kruskal-Wallis tests to compare the differences in PRS-IQ between cases, intrafamilial controls, and extrafamilial controls across cohorts. The difference in PRS-IQ between cohorts for each analysis group using the raw PRS-IQ (**a**) (Welch's p-value = 8.54e-06; Kruskal-Wallis p-value = 4.58e-05) was removed after scaling the PRS-IQ and accounting for ancestry PCs (**b**) (Welch's p-value = 0.38; Kruskal-Wallis p-value = 0.41).

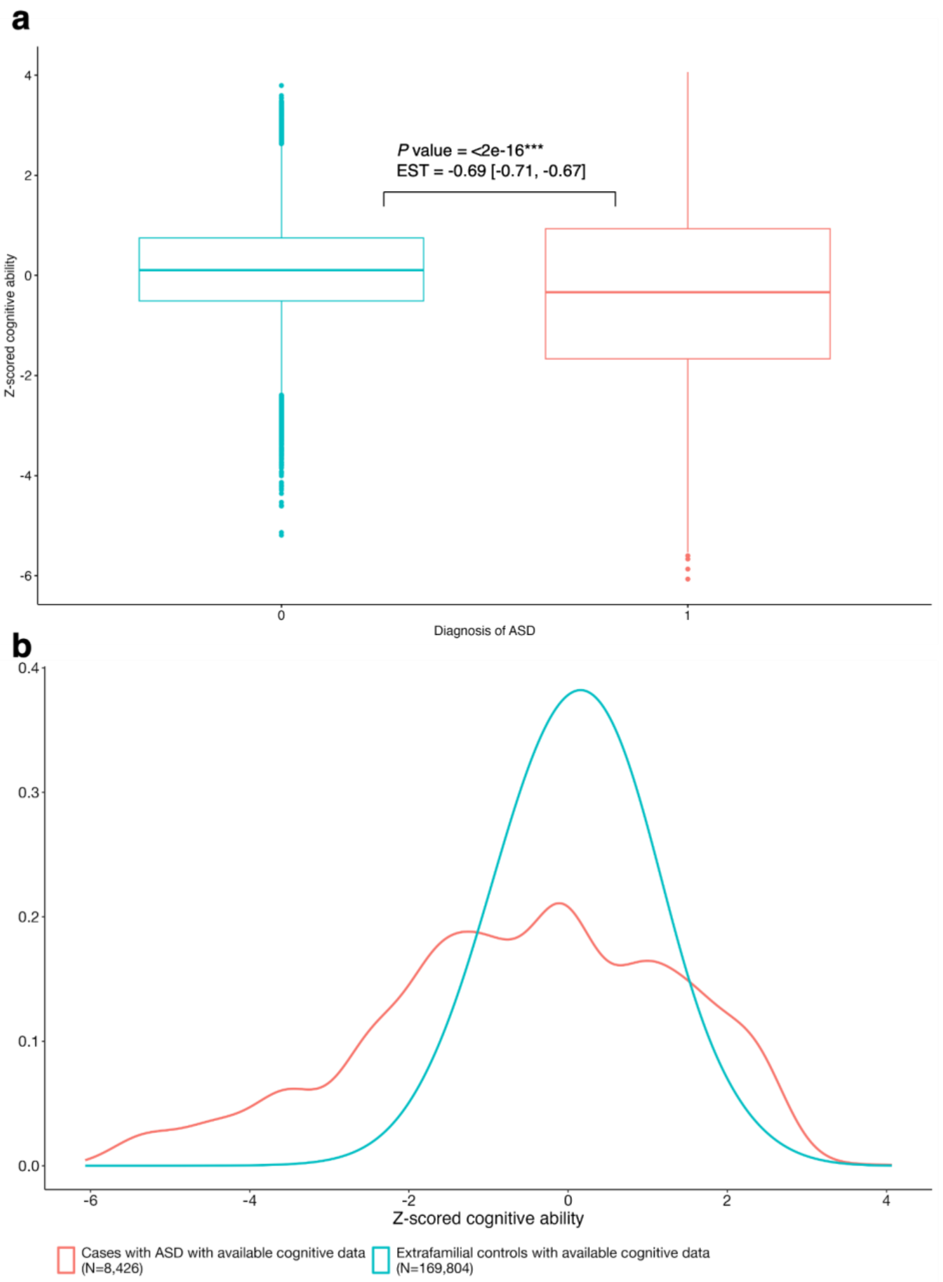

Figure S4. Distribution of scaled cognitive ability.
a) On average, the mean cognitive ability of subjects with ASD was 0.69 units lower than extrafamilial controls. *P* value was the result of a linear regression using ASD status as predictor, and cognitive ability as the outcome. The model included sex as a covariate. **b**) Density plot representing the distribution of scaled cognitive measures across cases with ASD (n=8,426) and extrafamilial controls (n=169,804) for whom cognitive ability data was available. The density of the curve was smoothed using a bandwidth selector of 0.4 for the Gaussian kernel density estimation.

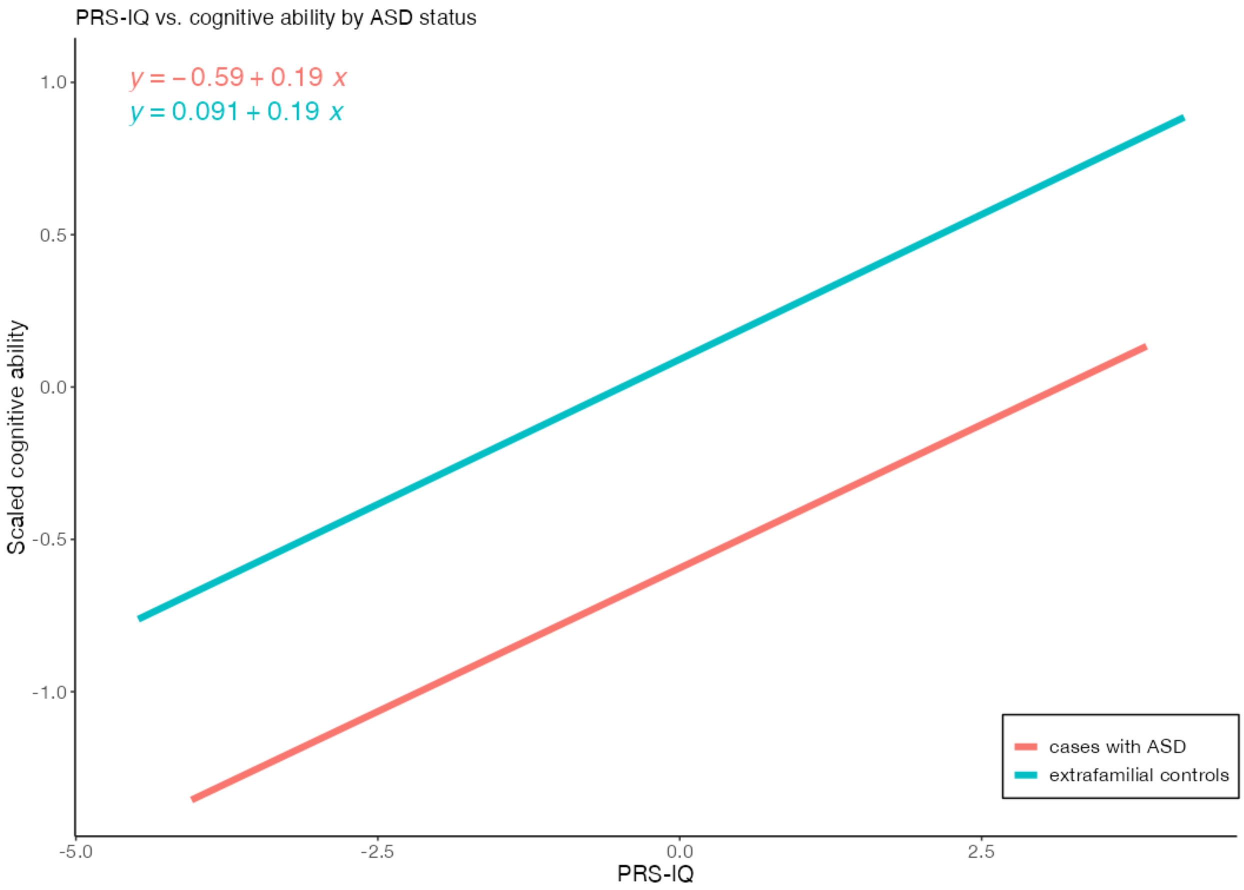

Figure S5. Correlation between PRS-IQ and cognitive ability in cases with ASD and controls.
Relationship between PRS-IQ and cognitive ability in cases with ASD in pink (n=8,426) and controls in blue (n=169,804). Each point represents an individual's PRS-IQ value plotted against their scaled cognitive ability. The trend line represents the linear relationship between cognitive ability as a function of PRS-IQ.

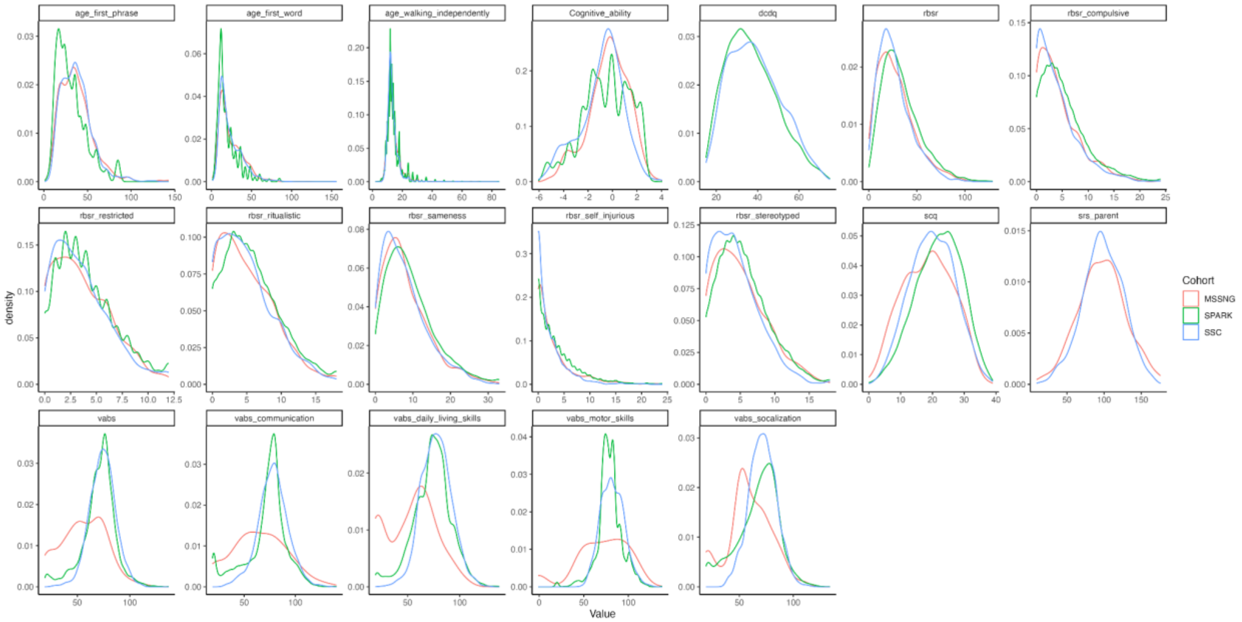

Figure S6. Distribution of raw continuous phenotypic measures among individuals with ASD, for the three ASD cohorts.

For a detailed description and abbreviation of the traits included in the study, see "Phenotypic measures" in Methods section of the manuscript.

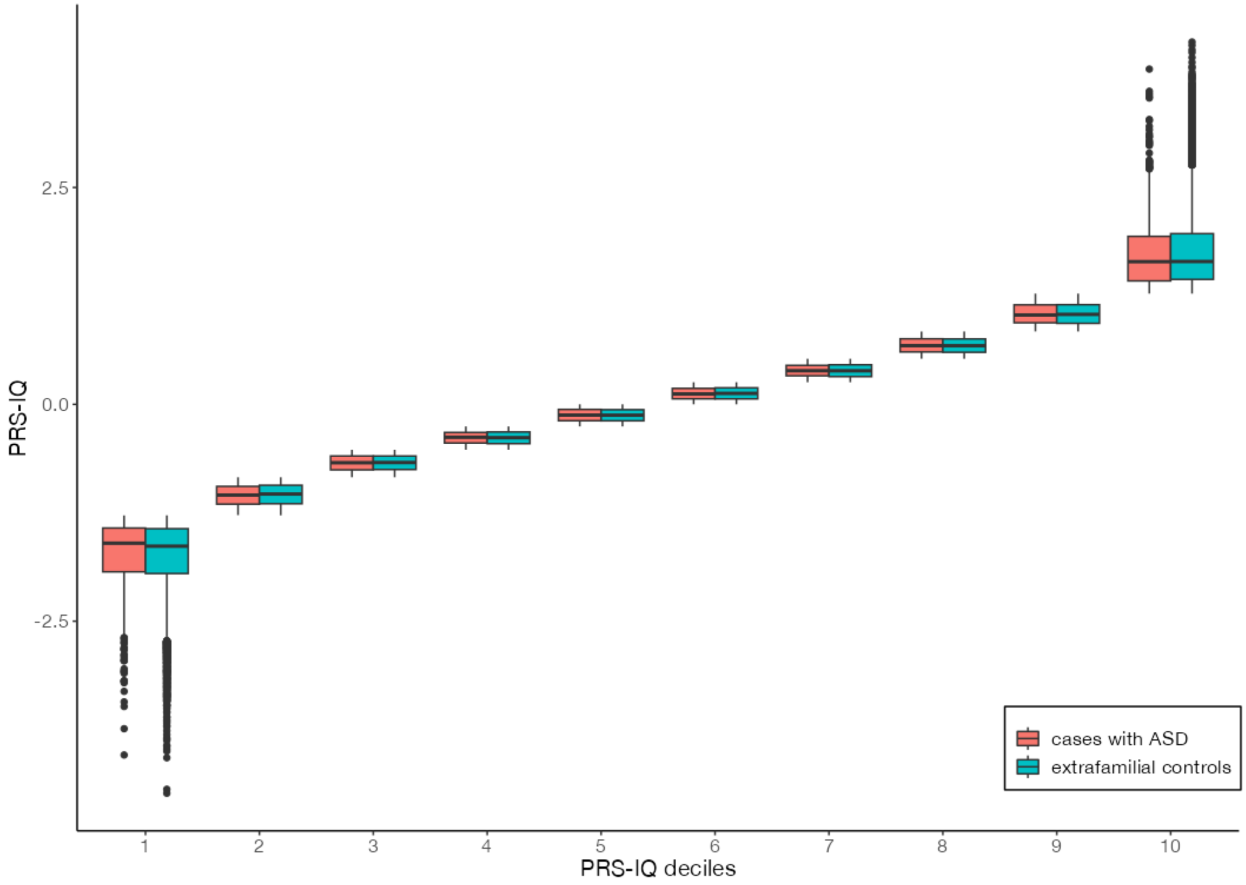

Figure S7. Boxplot illustrating the distribution of PRS-IQ across deciles for cases and controls. Distribution of PRS-IQ across each decile for cases (in pink) and extrafamilial controls (in blue). A Wilcoxon rank-sum test identified no statistical difference in the distribution of PRS-IQ between cases and extrafamilial controls across all deciles (p-values: decile 1: 0.266, decile 2: 0.151, decile 3: 0.967, decile 4: 0.663, decile 5: 0.53, decile 6: 0.346, decile 7: 0.81, decile 8: 0.52, decile 9: 0.965, decile 10: 0.246).

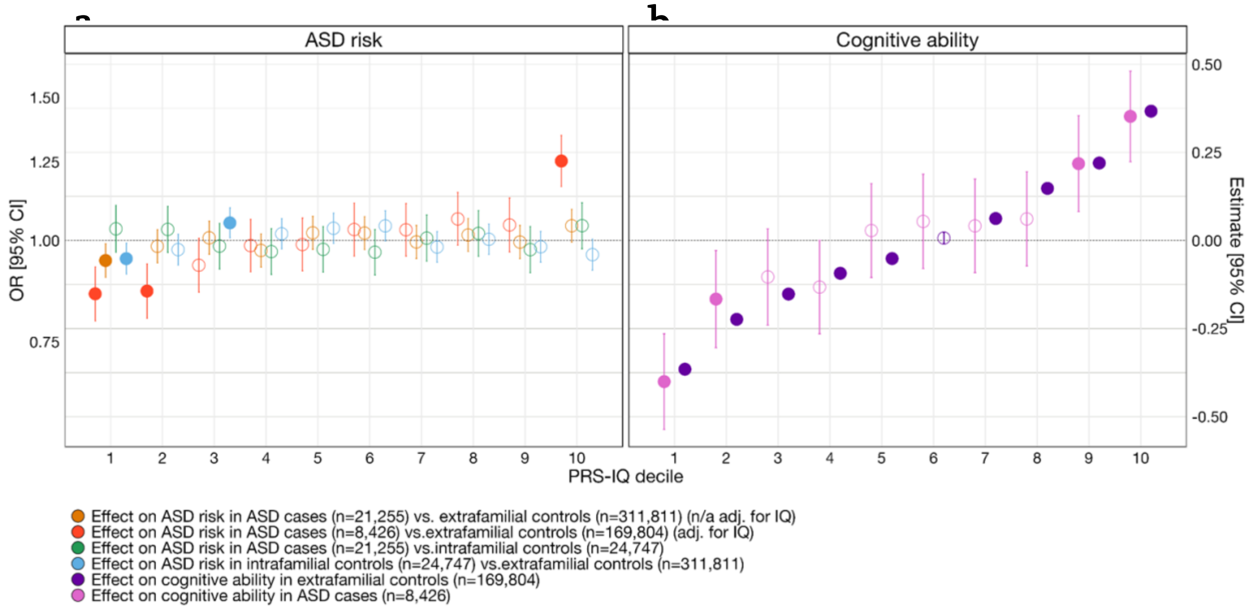

Figure S8. The effect of PRS-IQ deciles on ASD risk and cognitive ability across all analysis groups.
The estimate and 95% CI of each PRS-IQ decile on ASD risk and cognitive ability. Each regression accounted for the individual-level burden of deletions, duplications, and – when available – cognitive ability of individuals included in the model. **a**) Even after adjusting for the effects of cognitive ability (red points), a high PRS-IQ (10th decile) increases the risk for ASD, while a low PRS-IQ (1st, 2nd deciles) decreases the risk for ASD. Cases with ASD in the lowest PRS-IQ decile also have a significantly lower risk for ASD than their unaffected family members. **b**) A PRS-IQ below and above the 6th decile significantly decreases and increases cognitive ability in the general population, respectively. The effect of PRS-IQ on cognitive ability is the same in cases with ASD and in the general population (extrafamilial controls). Filled-in points represent statistically significant terms (P value ≤ 0.05 following FDR adjustment for multiple corrections). For detailed model results, see Table ST6.

Impact of PRS-IQ on ASD risk in carriers of CNVs encompassing highly intolerant genes

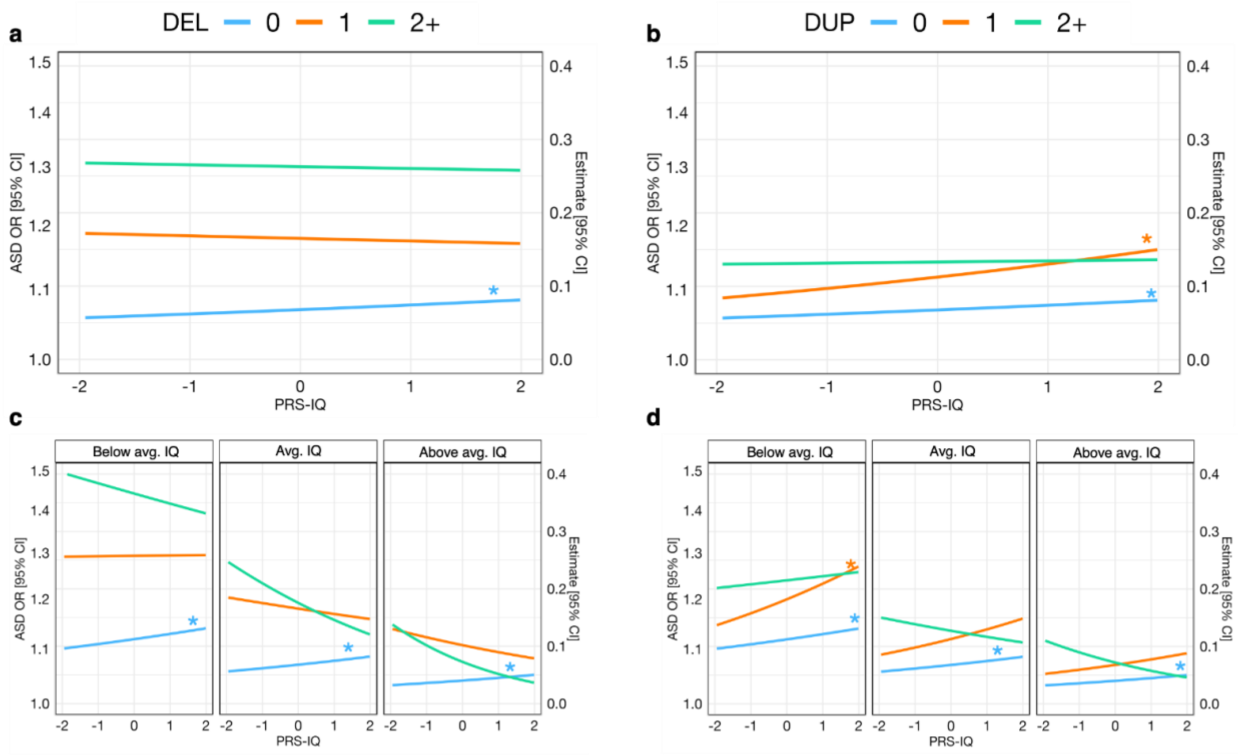

Impact of PRS-IQ on cognitive ability in carriers of CNVs encompassing highly intolerant genes

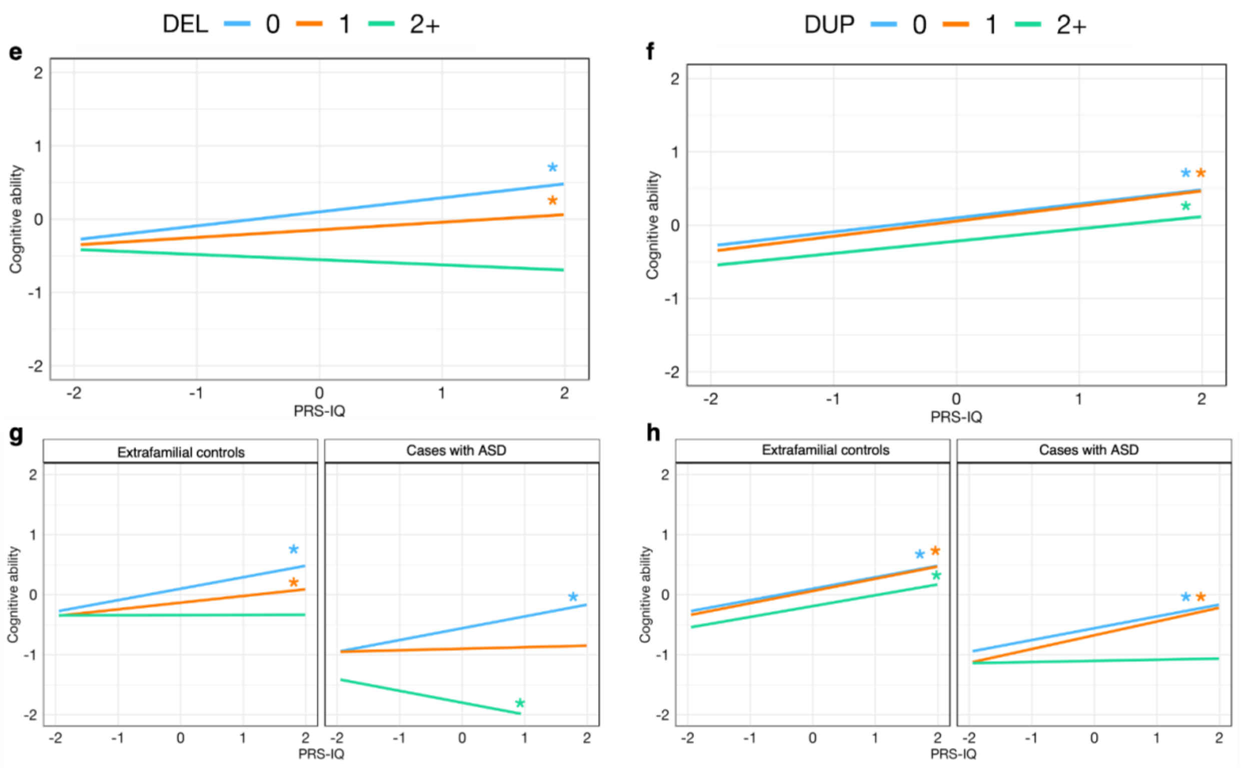

Figure S9. Deletions and duplications in highly intolerant genes modulate the impact of PRS-IQ on ASD risk and cognitive ability.

Deconstructing the significant negative interaction between CNV burden and PRS-IQ that was identified in Figure 1. These interaction plots highlight the impact of highly intolerant CNVs in attenuating the effect of PRS-IQ on ASD risk and cognitive ability. **a,b)** Interaction plot showing the effect of PRS-IQ on ASD risk as moderated by the number of highly intolerant CNVs carried by each individual. PRS-IQ has a positive linear impact on ASD risk in individuals who do not carry any highly-intolerant deletions (n_{cases}=8,170; $n_{controls}$=168,499) or duplications (n_{cases}=8,028; $n_{controls}$=166,003). Carrying 1 deletion reduces the impact of PRS-IQ on ASD (n_{cases}=162; $n_{controls}$=1,049), and the negative effect of PRS-IQ on ASD risk is exacerbated in carriers of ≥2 deletions (n_{cases}=94; $n_{controls}$=256). Carrying highly intolerant duplications attenuates, but to a lesser extent compared to deletions, the effect of PRS-IQ on ASD risk (n_{cases}=227; $n_{controls}$=2512 carrying one duplication, and n_{cases}=171; $n_{controls}$=1,289 carrying ≥2 duplications). **c, d**) Adding cognitive ability as the second moderator reveals that these trends remain consistent, regardless of the scaled cognitive ability (1 standard deviation below the mean (-0.97), mean (0.067), or 1 standard deviation above the mean (1.10)) of the samples. **e, f**) Interaction plot showing the effect of PRS-IQ on cognitive ability as moderated by the number of highly intolerant CNVs carried by each subject. Except for individuals that carry ≥2 highly intolerant deletions, PRS-IQ has a positive linear impact on cognitive ability. However, carrying highly-intolerant deletions attenuated the impact of PRS-IQ on cognitive ability. The attenuation of PRS-IQ in the presence of highly intolerant deletions (**g**) and duplications (**h**) was more pronounced in cases with ASD compared to extrafamilial controls. Asterisks (*) denote statistically significant estimates of PRS-IQ, interacting with its moderator variable(s) (*P* value ≤ 0.05 following FDR adjustment for multiple corrections). While not all regression lines are statistically significant, these interaction plots should be interpreted as descriptive trends between the genetic risk factors and outcomes. See Table ST4 and Table ST5 for a detailed number of carriers and regression model results.

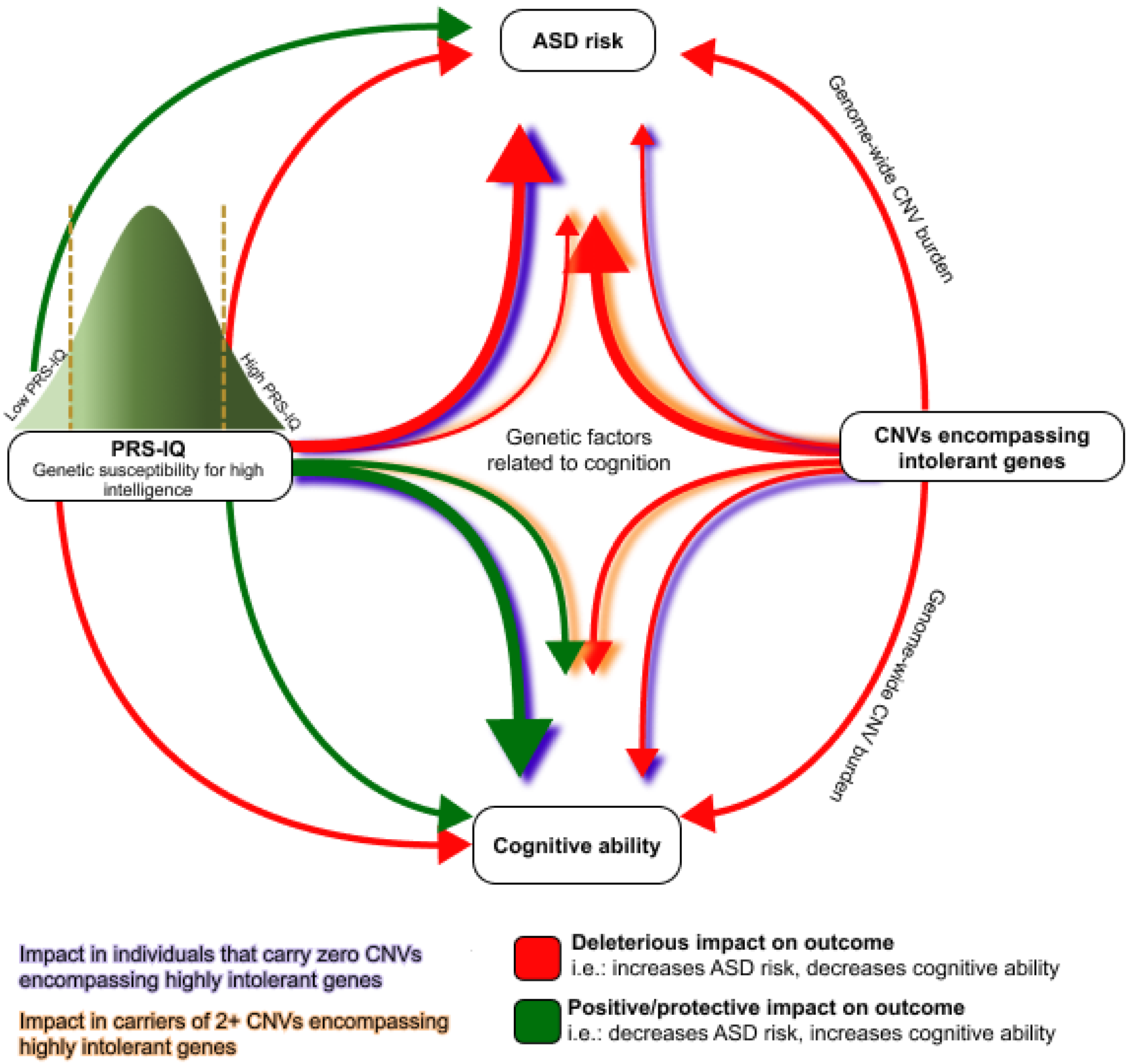

Figure S10. Summary of the impact of cognition-related genetic factors on ASD risk and cognitive ability. Representation of the main findings from this study. Of note, the impact of cognition-related genetic factors on ASD risk remains unchanged, even after adjusting for cognitive ability in the models. The thickness of the arrows denotes the relative impact of the variant class on the outcome. Our findings suggest that low PRS-IQ decreases ASD risk and decreases cognitive ability. Conversely, high PRS-IQ increases ASD risk and increases cognitive ability. The genome-wide burden of deletions and duplications increases the risk for ASD and decreases cognitive ability. Our preliminary findings suggest that ASD risk may be mostly driven by PRS-IQ among individuals who carry no CNVs in highly intolerant genes (purple highlight). Conversely, PRS-IQ may play a minimal role in ASD risk among individuals who carry ≥2 CNVs in highly intolerant genes (orange highlight). Future studies with larger samples are warranted to corroborate this complex interplay.
